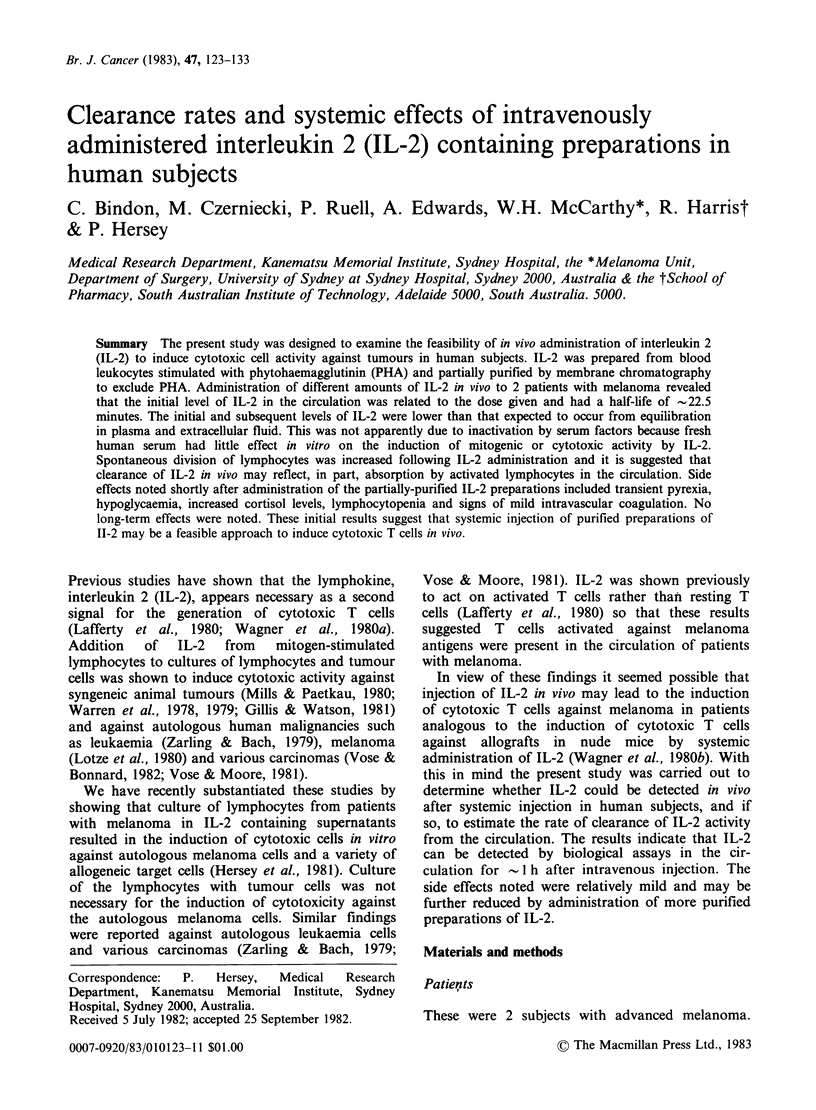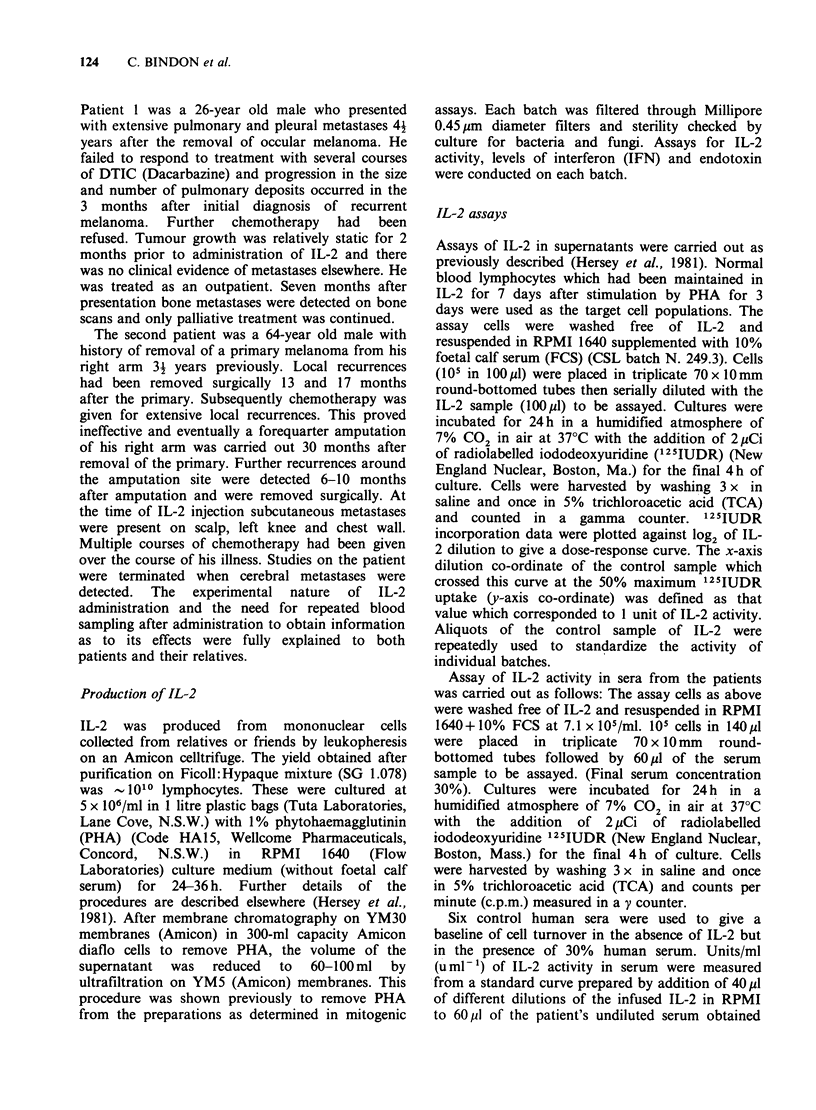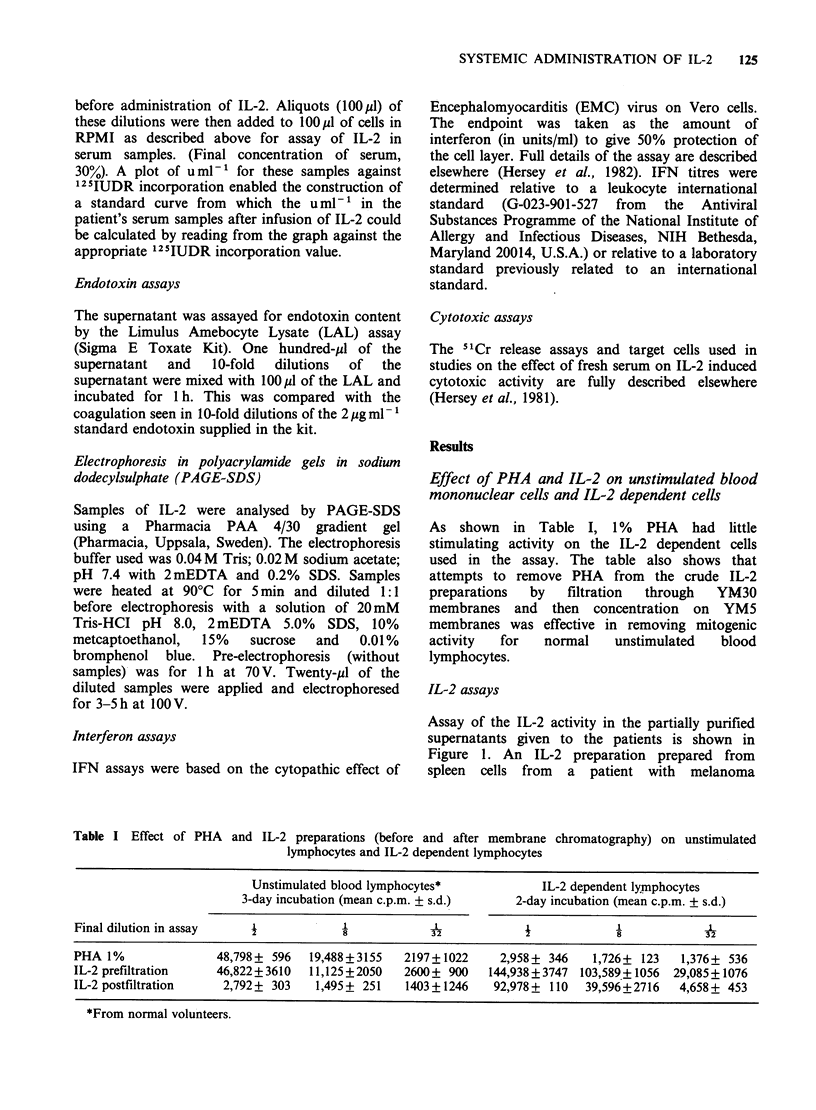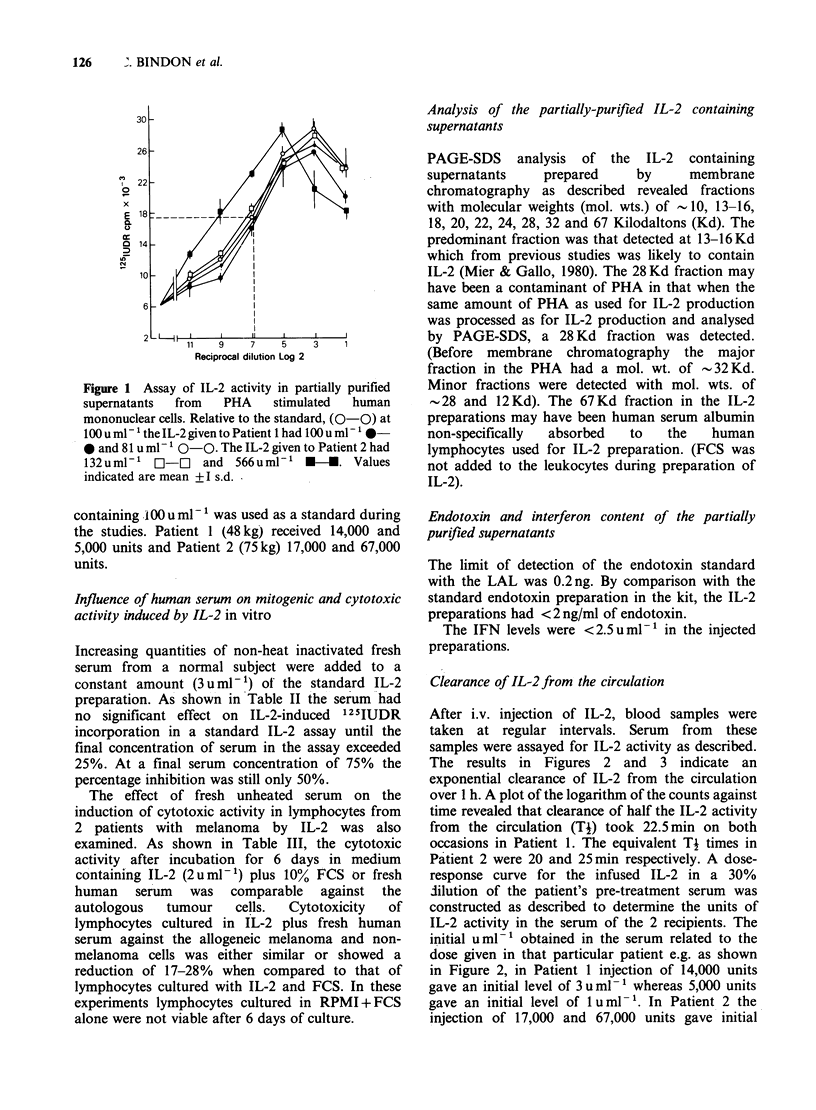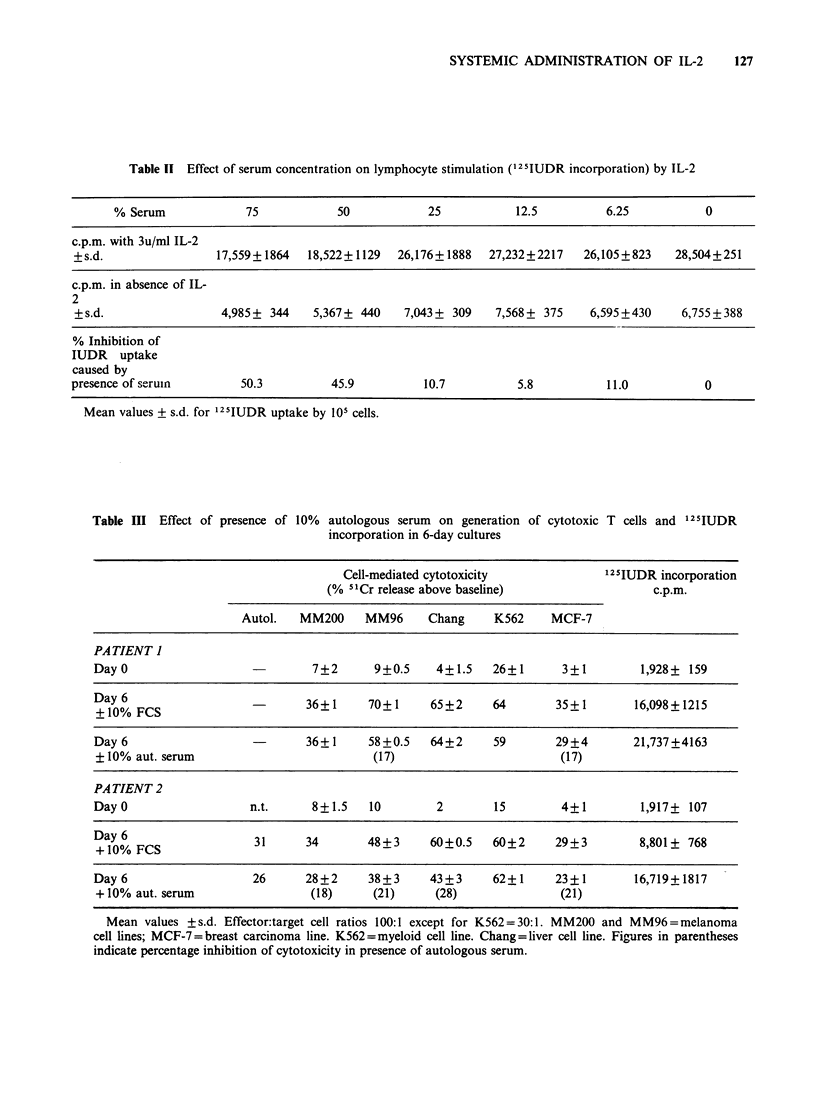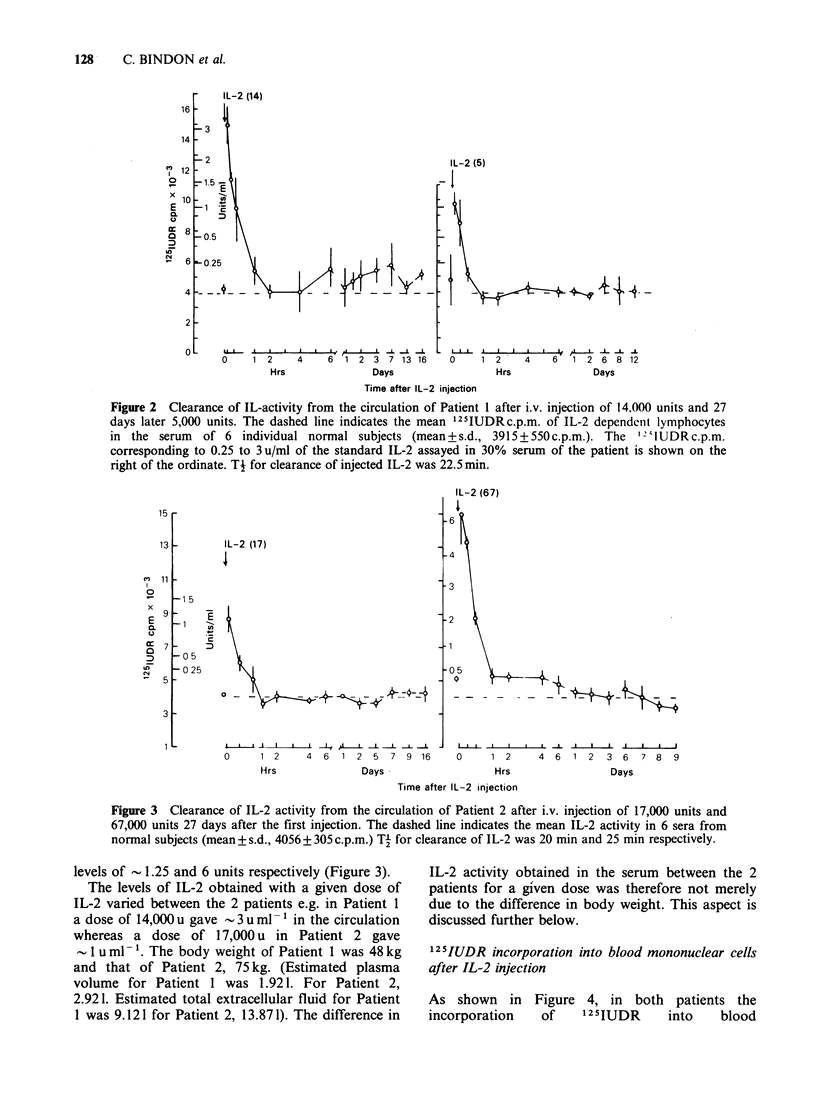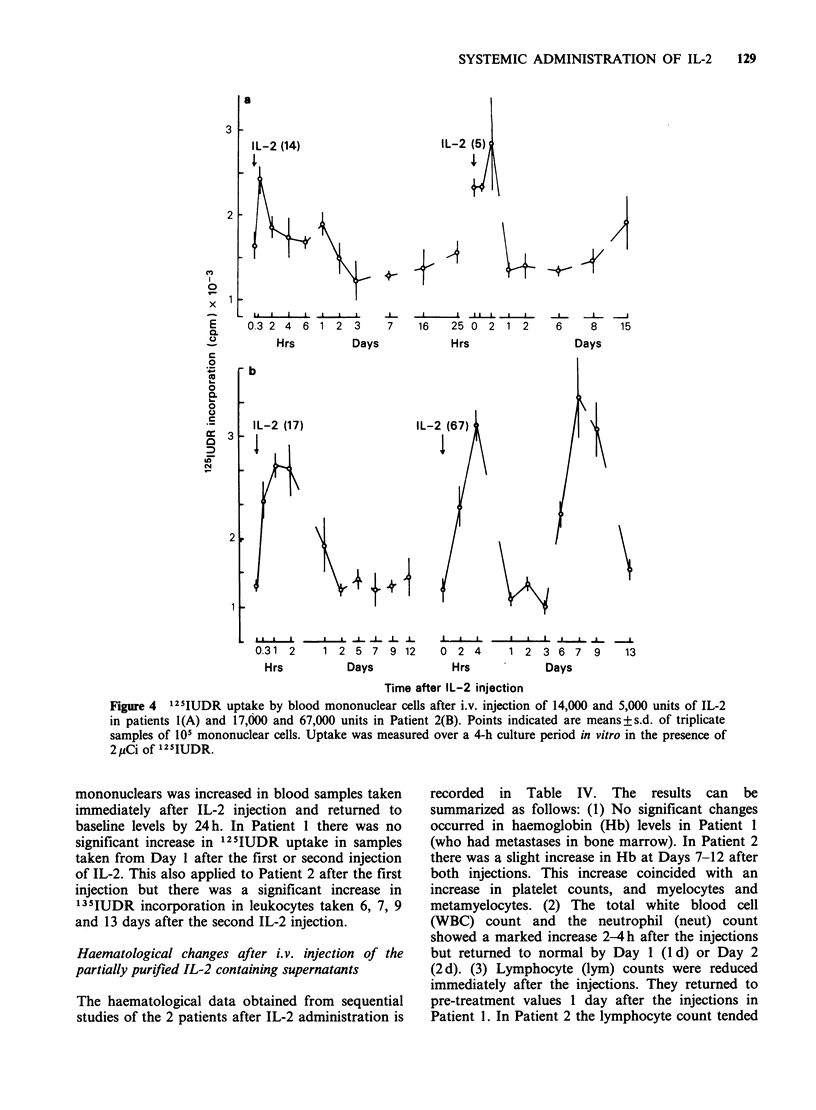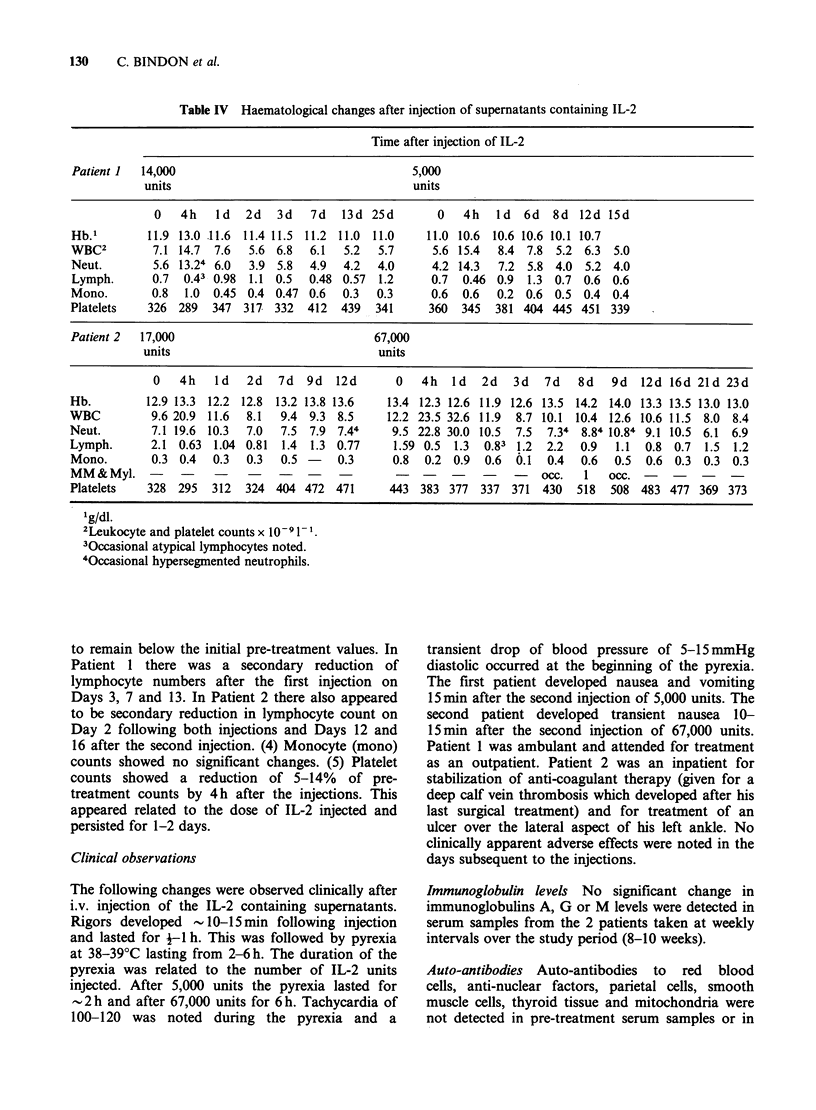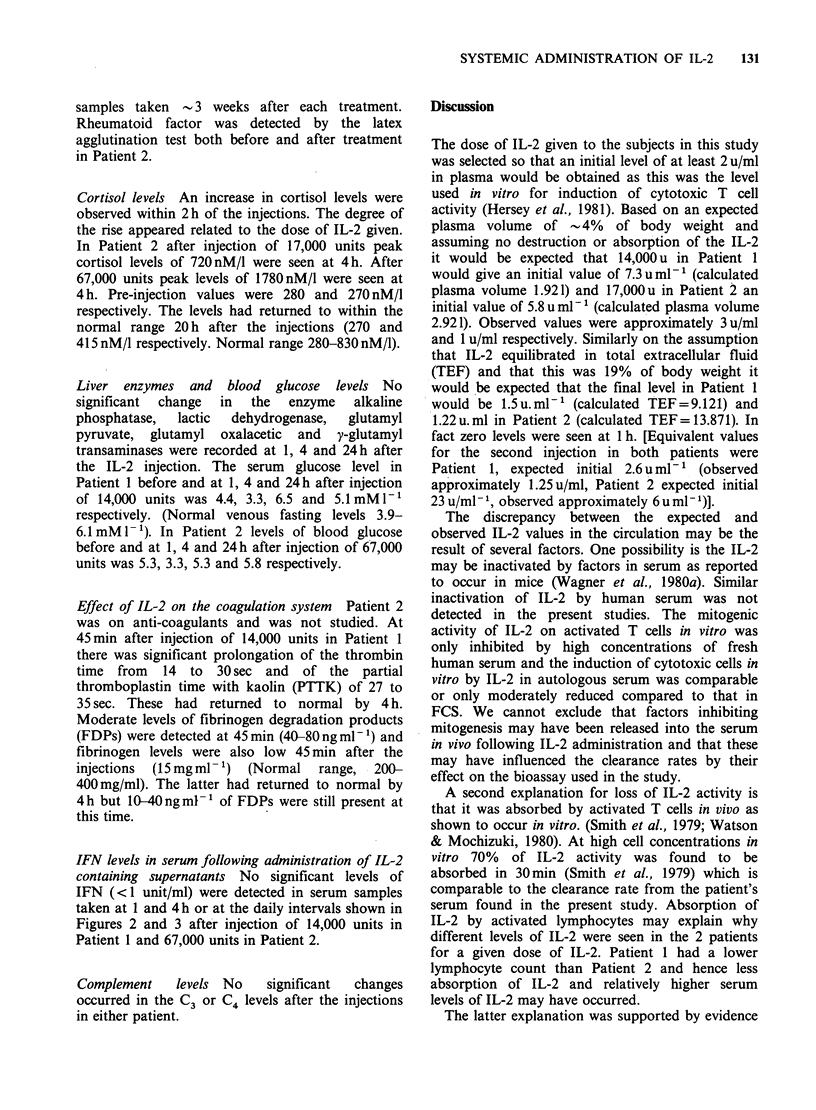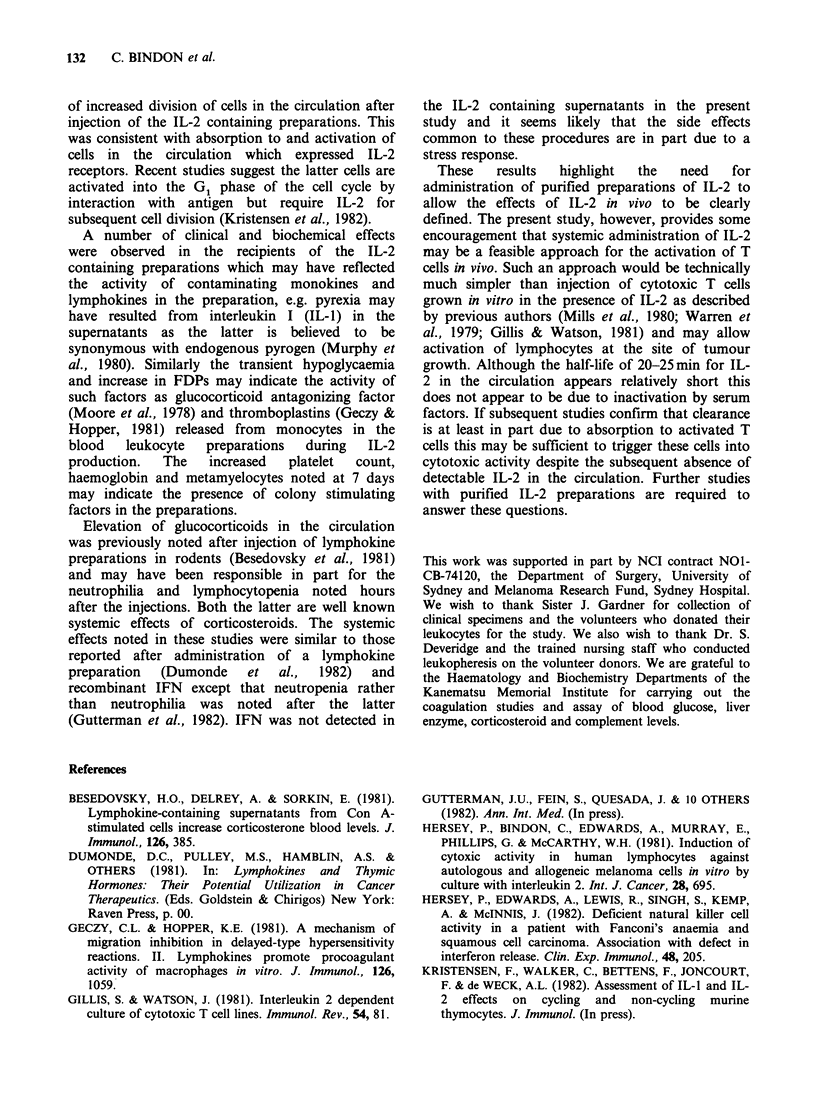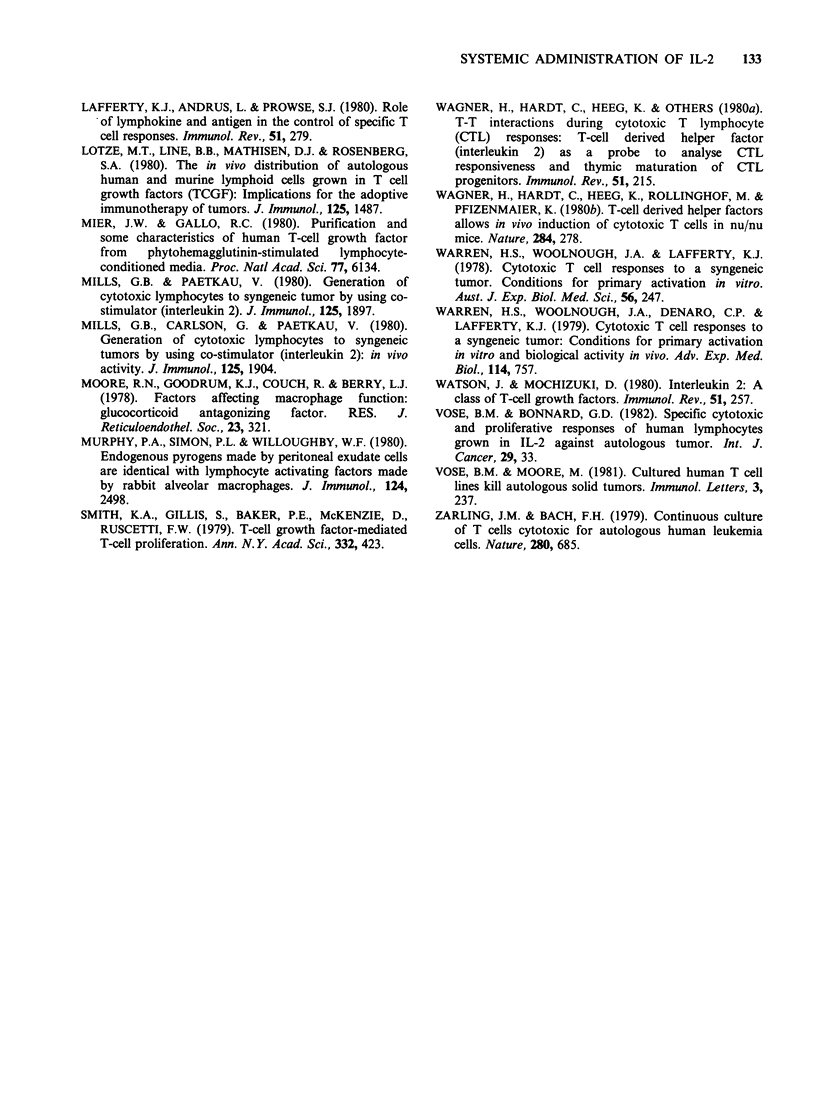# Clearance rates and systemic effects of intravenously administered interleukin 2 (IL-2) containing preparations in human subjects.

**DOI:** 10.1038/bjc.1983.15

**Published:** 1983-01

**Authors:** C. Bindon, M. Czerniecki, P. Ruell, A. Edwards, W. H. McCarthy, R. Harris, P. Hersey

## Abstract

The present study was designed to examine the feasibility of in vivo administration of interleukin 2 (IL-2) to induce cytotoxic cell activity against tumours in human subjects. IL-2 was prepared from blood leukocytes stimulated with phytohaemagglutinin (PHA) and partially purified by membrane chromatography to exclude PHA. Administration of different amounts of IL-2 in vivo to 2 patients with melanoma revealed that the initial level of IL-2 in the circulation was related to the dose given and had a half-life of approximately 22.5 minutes. The initial and subsequent levels of IL-2 were lower than that expected to occur from equilibration in plasma and extracellular fluid. This was not apparently due to inactivation by serum factors because fresh human serum had little effect in vitro on the induction of mitogenic or cytotoxic activity by IL-2. Spontaneous division of lymphocytes was increased following IL-2 administration and it is suggested that clearance of IL-2 in vivo may reflect, in part, absorption by activated lymphocytes in the circulation. Side effects noted shortly after administration of the partially-purified IL-2 preparations included transient pyrexia, hypoglycaemia, increased cortisol levels, lymphocytopenia and signs of mild intravascular coagulation. No long-term effects were noted. These initial results suggest that systemic injection of purified preparations of II-2 may be a feasible approach to induce cytotoxic T cells in vivo.


Br. J. Cancer (1983), 47, 123-133

### Clearance rates and systemic effects of intravenously administered interleukin 2 (IL-2) containing preparations in human subjects

C. Bindon, M. Czerniecki, P. Ruell, A. Edwards, W.H. McCarthy*, R. Harris† & P. Hersey

*Medical Research Department, Kanematsu Memorial Institute, Sydney Hospital, the *Melanoma Unit, Department of Surgery, University of Sydney at Sydney Hospital, Sydney 2000, Australia & the †School of Pharmacy, South Australian Institute of Technology, Adelaide 5000, South Australia. 5000.*

**Summary** The present study was designed to examine the feasibility of *in vivo* administration of interleukin 2 (IL-2) to induce cytotoxic cell activity against tumours in human subjects. IL-2 was prepared from blood leukocytes stimulated with phytohaemagglutinin (PHA) and partially purified by membrane chromatography to exclude PHA. Administration of different amounts of IL-2 *in vivo* to 2 patients with melanoma revealed that the initial level of IL-2 in the circulation was related to the dose given and had a half-life of ~22.5 minutes. The initial and subsequent levels of IL-2 were lower than that expected to occur from equilibration in plasma and extracellular fluid. This was not apparently due to inactivation by serum factors because fresh human serum had little effect *in vitro* on the induction of mitogenic or cytotoxic activity by IL-2. Spontaneous division of lymphocytes was increased following IL-2 administration and it is suggested that clearance of IL-2 *in vivo* may reflect, in part, absorption by activated lymphocytes in the circulation. Side effects noted shortly after administration of the partially-purified IL-2 preparations included transient pyrexia, hypoglycaemia, increased cortisol levels, lymphocytopenia and signs of mild intravascular coagulation. No long-term effects were noted. These initial results suggest that systemic injection of purified preparations of Il-2 may be a feasible approach to induce cytotoxic T cells *in vivo*.

Previous studies have shown that the lymphokine, interleukin 2 (IL-2), appears necessary as a second signal for the generation of cytotoxic T cells (Lafferty *et al.*, 1980; Wagner *et al.*, 1980*a*). Addition of IL-2 from mitogen-stimulated lymphocytes to cultures of lymphocytes and tumour cells was shown to induce cytotoxic activity against syngeneic animal tumours (Mills & Paetkau, 1980; Warren *et al.*, 1978, 1979; Gillis & Watson, 1981) and against autologous human malignancies such as leukaemia (Zarling & Bach, 1979), melanoma (Lotze *et al.*, 1980) and various carcinomas (Vose & Bonnard, 1982; Vose & Moore, 1981).

We have recently substantiated these studies by showing that culture of lymphocytes from patients with melanoma in IL-2 containing supernatants resulted in the induction of cytotoxic cells *in vitro* against autologous melanoma cells and a variety of allogeneic target cells (Hersey *et al.*, 1981). Culture of the lymphocytes with tumour cells was not necessary for the induction of cytotoxicity against the autologous melanoma cells. Similar findings were reported against autologous leukaemia cells and various carcinomas (Zarling & Bach, 1979; Vose & Moore, 1981). IL-2 was shown previously to act on activated T cells rather than resting T cells (Lafferty *et al.*, 1980) so that these results suggested T cells activated against melanoma antigens were present in the circulation of patients with melanoma.

In view of these findings it seemed possible that injection of IL-2 *in vivo* may lead to the induction of cytotoxic T cells against melanoma in patients analogous to the induction of cytotoxic T cells against allografts in nude mice by systemic administration of IL-2 (Wagner *et al.*, 1980*b*). With this in mind the present study was carried out to determine whether IL-2 could be detected *in vivo* after systemic injection in human subjects, and if so, to estimate the rate of clearance of IL-2 activity from the circulation. The results indicate that IL-2 can be detected by biological assays in the circulation for ~1 h after intravenous injection. The side effects noted were relatively mild and may be further reduced by administration of more purified preparations of IL-2.

### Materials and methods

### *Patients*

These were 2 subjects with advanced melanoma.

Correspondence: P. Hersey, Medical Research Department, Kanematsu Memorial Institute, Sydney Hospital, Sydney 2000, Australia.
Received 5 July 1982; accepted 25 September 1982.

0007-0920/83/010123-11 $01.00

© The Macmillan Press Ltd., 1983

Patient 1 was a 26-year old male who presented with extensive pulmonary and pleural metastases 4½ years after the removal of occular melanoma. He failed to respond to treatment with several courses of DTIC (Dacarbazine) and progression in the size and number of pulmonary deposits occurred in the 3 months after initial diagnosis of recurrent melanoma. Further chemotherapy had been refused. Tumour growth was relatively static for 2 months prior to administration of IL-2 and there was no clinical evidence of metastases elsewhere. He was treated as an outpatient. Seven months after presentation bone metastases were detected on bone scans and only palliative treatment was continued.

The second patient was a 64-year old male with history of removal of a primary melanoma from his right arm 3½ years previously. Local recurrences had been removed surgically 13 and 17 months after the primary. Subsequently chemotherapy was given for extensive local recurrences. This proved ineffective and eventually a forequarter amputation of his right arm was carried out 30 months after removal of the primary. Further recurrences around the amputation site were detected 6–10 months after amputation and were removed surgically. At the time of IL-2 injection subcutaneous metastases were present on scalp, left knee and chest wall. Multiple courses of chemotherapy had been given over the course of his illness. Studies on the patient were terminated when cerebral metastases were detected. The experimental nature of IL-2 administration and the need for repeated blood sampling after administration to obtain information as to its effects were fully explained to both patients and their relatives.

*Production of IL-2*

IL-2 was produced from mononuclear cells collected from relatives or friends by leukopheresis on an Amicon celltrifuge. The yield obtained after purification on Ficoll:Hypaque mixture (SG 1.078) was $\sim 10^{10}$ lymphocytes. These were cultured at $5 \times 10^6$/ml in 1 litre plastic bags (Tuta Laboratories, Lane Cove, N.S.W.) with 1% phytohaemagglutinin (PHA) (Code HA15, Wellcome Pharmaceuticals, Concord, N.S.W.) in RPMI 1640 (Flow Laboratories) culture medium (without foetal calf serum) for 24–36 h. Further details of the procedures are described elsewhere (Hersey *et al.*, 1981). After membrane chromatography on YM30 membranes (Amicon) in 300-ml capacity Amicon diaflo cells to remove PHA, the volume of the supernatant was reduced to 60–100 ml by ultrafiltration on YM5 (Amicon) membranes. This procedure was shown previously to remove PHA from the preparations as determined in mitogenic assays. Each batch was filtered through Millipore 0.45 μm diameter filters and sterility checked by culture for bacteria and fungi. Assays for IL-2 activity, levels of interferon (IFN) and endotoxin were conducted on each batch.

*IL-2 assays*

Assays of IL-2 in supernatants were carried out as previously described (Hersey *et al.*, 1981). Normal blood lymphocytes which had been maintained in IL-2 for 7 days after stimulation by PHA for 3 days were used as the target cell populations. The assay cells were washed free of IL-2 and resuspended in RPMI 1640 supplemented with 10% foetal calf serum (FCS) (CSL batch N. 249.3). Cells ($10^5$ in 100 μl) were placed in triplicate 70 × 10 mm round-bottomed tubes then serially diluted with the IL-2 sample (100 μl) to be assayed. Cultures were incubated for 24 h in a humidified atmosphere of 7% $CO_2$ in air at 37°C with the addition of 2 μCi of radiolabelled iododeoxyuridine ($^{125}$IUDR) (New England Nuclear, Boston, Ma.) for the final 4 h of culture. Cells were harvested by washing 3 × in saline and once in 5% trichloroacetic acid (TCA) and counted in a gamma counter. $^{125}$IUDR incorporation data were plotted against $\log_2$ of IL-2 dilution to give a dose-response curve. The $x$-axis dilution co-ordinate of the control sample which crossed this curve at the 50% maximum $^{125}$IUDR uptake ($y$-axis co-ordinate) was defined as that value which corresponded to 1 unit of IL-2 activity. Aliquots of the control sample of IL-2 were repeatedly used to standardize the activity of individual batches.

Assay of IL-2 activity in sera from the patients was carried out as follows: The assay cells as above were washed free of IL-2 and resuspended in RPMI 1640 + 10% FCS at $7.1 \times 10^5$/ml. $10^5$ cells in 140 μl were placed in triplicate 70 × 10 mm round-bottomed tubes followed by 60 μl of the serum sample to be assayed. (Final serum concentration 30%). Cultures were incubated for 24 h in a humidified atmosphere of 7% $CO_2$ in air at 37°C with the addition of 2 μCi of radiolabelled iododeoxyuridine $^{125}$IUDR (New England Nuclear, Boston, Mass.) for the final 4 h of culture. Cells were harvested by washing 3 × in saline and once in 5% trichloroacetic acid (TCA) and counts per minute (c.p.m.) measured in a γ counter.

Six control human sera were used to give a baseline of cell turnover in the absence of IL-2 but in the presence of 30% human serum. Units/ml (u $ml^{-1}$) of IL-2 activity in serum were measured from a standard curve prepared by addition of 40 μl of different dilutions of the infused IL-2 in RPMI to 60 μl of the patient's undiluted serum obtained before administration of IL-2. Aliquots (100 μl) of these dilutions were then added to 100 μl of cells in RPMI as described above for assay of IL-2 in serum samples. (Final concentration of serum, 30%). A plot of $u\,ml^{-1}$ for these samples against $^{125}$IUDR incorporation enabled the construction of a standard curve from which the $u\,ml^{-1}$ in the patient's serum samples after infusion of IL-2 could be calculated by reading from the graph against the appropriate $^{125}$IUDR incorporation value.

*Endotoxin assays*

The supernatant was assayed for endotoxin content by the Limulus Amebocyte Lysate (LAL) assay (Sigma E Toxate Kit). One hundred-μl of the supernatant and 10-fold dilutions of the supernatant were mixed with 100 μl of the LAL and incubated for 1 h. This was compared with the coagulation seen in 10-fold dilutions of the $2\,\mu g\,ml^{-1}$ standard endotoxin supplied in the kit.

*Electrophoresis in polyacrylamide gels in sodium dodecylsulphate (PAGE-SDS)*

Samples of IL-2 were analysed by PAGE-SDS using a Pharmacia PAA 4/30 gradient gel (Pharmacia, Uppsala, Sweden). The electrophoresis buffer used was 0.04 M Tris; 0.02 M sodium acetate; pH 7.4 with 2 mEDTA and 0.2% SDS. Samples were heated at 90°C for 5 min and diluted 1:1 before electrophoresis with a solution of 20 mM Tris-HCI pH 8.0, 2 mEDTA 5.0% SDS, 10% metcaptoethanol, 15% sucrose and 0.01% bromphenol blue. Pre-electrophoresis (without samples) was for 1 h at 70 V. Twenty-μl of the diluted samples were applied and electrophoresed for 3–5 h at 100 V.

*Interferon assays*

IFN assays were based on the cytopathic effect of Encephalomyocarditis (EMC) virus on Vero cells. The endpoint was taken as the amount of interferon (in units/ml) to give 50% protection of the cell layer. Full details of the assay are described elsewhere (Hersey *et al.*, 1982). IFN titres were determined relative to a leukocyte international standard (G-023-901-527 from the Antiviral Substances Programme of the National Institute of Allergy and Infectious Diseases, NIH Bethesda, Maryland 20014, U.S.A.) or relative to a laboratory standard previously related to an international standard.

*Cytotoxic assays*

The $^{51}$Cr release assays and target cells used in studies on the effect of fresh serum on IL-2 induced cytotoxic activity are fully described elsewhere (Hersey *et al.*, 1981).

### Results

### *Effect of PHA and IL-2 on unstimulated blood mononuclear cells and IL-2 dependent cells*

As shown in Table I, 1% PHA had little stimulating activity on the IL-2 dependent cells used in the assay. The table also shows that attempts to remove PHA from the crude IL-2 preparations by filtration through YM30 membranes and then concentration on YM5 membranes was effective in removing mitogenic activity for normal unstimulated blood lymphocytes.

*IL-2 assays*

Assay of the IL-2 activity in the partially purified supernatants given to the patients is shown in Figure 1. An IL-2 preparation prepared from spleen cells from a patient with melanoma

**Table I** Effect of PHA and IL-2 preparations (before and after membrane chromatography) on unstimulated lymphocytes and IL-2 dependent lymphocytes

| | Unstimulated blood lymphocytes* 3-day incubation (mean c.p.m. ± s.d.) | | | IL-2 dependent lymphocytes 2-day incubation (mean c.p.m. ± s.d.) | | |
|---|---|---|---|---|---|---|
| Final dilution in assay | ½ | ⅛ | 1/32 | ½ | ⅛ | 1/32 |
| PHA 1% | 48,798 ± 596 | 19,488 ± 3155 | 2197 ± 1022 | 2,958 ± 346 | 1,726 ± 123 | 1,376 ± 536 |
| IL-2 prefiltration | 46,822 ± 3610 | 11,125 ± 2050 | 2600 ± 900 | 144,938 ± 3747 | 103,589 ± 1056 | 29,085 ± 1076 |
| IL-2 postfiltration | 2,792 ± 303 | 1,495 ± 251 | 1403 ± 1246 | 92,978 ± 110 | 39,596 ± 2716 | 4,658 ± 453 |

*From normal volunteers.

**Figure 1** Assay of IL-2 activity in partially purified supernatants from PHA stimulated human mononuclear cells. Relative to the standard, (○—○) at 100 u ml$^{-1}$ the IL-2 given to Patient 1 had 100 u ml$^{-1}$ ●—● and 81 u ml$^{-1}$ ○—○. The IL-2 given to Patient 2 had 132 u ml$^{-1}$ □—□ and 566 u ml$^{-1}$ ■—■. Values indicated are mean ±1 s.d.

containing 100 u ml$^{-1}$ was used as a standard during the studies. Patient 1 (48 kg) received 14,000 and 5,000 units and Patient 2 (75 kg) 17,000 and 67,000 units.

*Influence of human serum on mitogenic and cytotoxic activity induced by IL-2* in vitro

Increasing quantities of non-heat inactivated fresh serum from a normal subject were added to a constant amount (3 u ml$^{-1}$) of the standard IL-2 preparation. As shown in Table II the serum had no significant effect on IL-2-induced $^{125}$IUDR incorporation in a standard IL-2 assay until the final concentration of serum in the assay exceeded 25%. At a final serum concentration of 75% the percentage inhibition was still only 50%.

The effect of fresh unheated serum on the induction of cytotoxic activity in lymphocytes from 2 patients with melanoma by IL-2 was also examined. As shown in Table III, the cytotoxic activity after incubation for 6 days in medium containing IL-2 (2 u ml$^{-1}$) plus 10% FCS or fresh human serum was comparable against the autologous tumour cells. Cytotoxicity of lymphocytes cultured in IL-2 plus fresh human serum against the allogeneic melanoma and non-melanoma cells was either similar or showed a reduction of 17–28% when compared to that of lymphocytes cultured with IL-2 and FCS. In these experiments lymphocytes cultured in RPMI + FCS alone were not viable after 6 days of culture.

*Analysis of the partially-purified IL-2 containing supernatants*

PAGE-SDS analysis of the IL-2 containing supernatants prepared by membrane chromatography as described revealed fractions with molecular weights (mol. wts.) of ~10, 13–16, 18, 20, 22, 24, 28, 32 and 67 Kilodaltons (Kd). The predominant fraction was that detected at 13–16 Kd which from previous studies was likely to contain IL-2 (Mier & Gallo, 1980). The 28 Kd fraction may have been a contaminant of PHA in that when the same amount of PHA as used for IL-2 production was processed as for IL-2 production and analysed by PAGE-SDS, a 28 Kd fraction was detected. (Before membrane chromatography the major fraction in the PHA had a mol. wt. of ~32 Kd. Minor fractions were detected with mol. wts. of ~28 and 12 Kd). The 67 Kd fraction in the IL-2 preparations may have been human serum albumin non-specifically absorbed to the human lymphocytes used for IL-2 preparation. (FCS was not added to the leukocytes during preparation of IL-2).

*Endotoxin and interferon content of the partially purified supernatants*

The limit of detection of the endotoxin standard with the LAL was 0.2 ng. By comparison with the standard endotoxin preparation in the kit, the IL-2 preparations had <2 ng/ml of endotoxin.

The IFN levels were <2.5 u ml$^{-1}$ in the injected preparations.

*Clearance of IL-2 from the circulation*

After i.v. injection of IL-2, blood samples were taken at regular intervals. Serum from these samples were assayed for IL-2 activity as described. The results in Figures 2 and 3 indicate an exponential clearance of IL-2 from the circulation over 1 h. A plot of the logarithm of the counts against time revealed that clearance of half the IL-2 activity from the circulation (T½) took 22.5 min on both occasions in Patient 1. The equivalent T½ times in Patient 2 were 20 and 25 min respectively. A dose-response curve for the infused IL-2 in a 30% dilution of the patient's pre-treatment serum was constructed as described to determine the units of IL-2 activity in the serum of the 2 recipients. The initial u ml$^{-1}$ obtained in the serum related to the dose given in that particular patient e.g. as shown in Figure 2, in Patient 1 injection of 14,000 units gave an initial level of 3 u ml$^{-1}$ whereas 5,000 units gave an initial level of 1 u ml$^{-1}$. In Patient 2 the injection of 17,000 and 67,000 units gave initial

**Table II** Effect of serum concentration on lymphocyte stimulation ($^{125}$IUDR incorporation) by IL-2

| % Serum | 75 | 50 | 25 | 12.5 | 6.25 | 0 |
|---|---|---|---|---|---|---|
| c.p.m. with 3u/ml IL-2 ±s.d. | 17,559±1864 | 18,522±1129 | 26,176±1888 | 27,232±2217 | 26,105±823 | 28,504±251 |
| c.p.m. in absence of IL-2 ±s.d. | 4,985± 344 | 5,367± 440 | 7,043± 309 | 7,568± 375 | 6,595±430 | 6,755±388 |
| % Inhibition of IUDR uptake caused by presence of serum | 50.3 | 45.9 | 10.7 | 5.8 | 11.0 | 0 |

Mean values ± s.d. for $^{125}$IUDR uptake by $10^5$ cells.

**Table III** Effect of presence of 10% autologous serum on generation of cytotoxic T cells and $^{125}$IUDR incorporation in 6-day cultures

| | Cell-mediated cytotoxicity (% $^{51}$Cr release above baseline) | | | | | | $^{125}$IUDR incorporation c.p.m. |
|---|---|---|---|---|---|---|---|
| | Autol. | MM200 | MM96 | Chang | K562 | MCF-7 | |
| *PATIENT 1* | | | | | | | |
| Day 0 | — | 7±2 | 9±0.5 | 4±1.5 | 26±1 | 3±1 | 1,928± 159 |
| Day 6 ±10% FCS | — | 36±1 | 70±1 | 65±2 | 64 | 35±1 | 16,098±1215 |
| Day 6 ±10% aut. serum | — | 36±1 | 58±0.5 (17) | 64±2 | 59 | 29±4 (17) | 21,737±4163 |
| *PATIENT 2* | | | | | | | |
| Day 0 | n.t. | 8±1.5 | 10 | 2 | 15 | 4±1 | 1,917± 107 |
| Day 6 +10% FCS | 31 | 34 | 48±3 | 60±0.5 | 60±2 | 29±3 | 8,801± 768 |
| Day 6 +10% aut. serum | 26 | 28±2 (18) | 38±3 (21) | 43±3 (28) | 62±1 | 23±1 (21) | 16,719±1817 |

Mean values ±s.d. Effector:target cell ratios 100:1 except for K562=30:1. MM200 and MM96=melanoma cell lines; MCF-7=breast carcinoma line. K562=myeloid cell line. Chang=liver cell line. Figures in parentheses indicate percentage inhibition of cytotoxicity in presence of autologous serum.

**Figure 2** Clearance of IL-activity from the circulation of Patient 1 after i.v. injection of 14,000 units and 27 days later 5,000 units. The dashed line indicates the mean $^{125}$IUDR c.p.m. of IL-2 dependent lymphocytes in the serum of 6 individual normal subjects (mean$\pm$s.d., 3915$\pm$550 c.p.m.). The $^{125}$IUDR c.p.m. corresponding to 0.25 to 3 u/ml of the standard IL-2 assayed in 30% serum of the patient is shown on the right of the ordinate. T½ for clearance of injected IL-2 was 22.5 min.

**Figure 3** Clearance of IL-2 activity from the circulation of Patient 2 after i.v. injection of 17,000 units and 67,000 units 27 days after the first injection. The dashed line indicates the mean IL-2 activity in 6 sera from normal subjects (mean$\pm$s.d., 4056$\pm$305 c.p.m.) T½ for clearance of IL-2 was 20 min and 25 min respectively.

levels of ~1.25 and 6 units respectively (Figure 3).

The levels of IL-2 obtained with a given dose of IL-2 varied between the 2 patients e.g. in Patient 1 a dose of 14,000 u gave ~3 u ml$^{-1}$ in the circulation whereas a dose of 17,000 u in Patient 2 gave ~1 u ml$^{-1}$. The body weight of Patient 1 was 48 kg and that of Patient 2, 75 kg. (Estimated plasma volume for Patient 1 was 1.92 l. For Patient 2, 2.92 l. Estimated total extracellular fluid for Patient 1 was 9.12 l for Patient 2, 13.87 l). The difference in IL-2 activity obtained in the serum between the 2 patients for a given dose was therefore not merely due to the difference in body weight. This aspect is discussed further below.

### *$^{125}$IUDR incorporation into blood mononuclear cells after IL-2 injection*

As shown in Figure 4, in both patients the incorporation of $^{125}$IUDR into blood

**Figure 4** $^{125}$IUDR uptake by blood mononuclear cells after i.v. injection of 14,000 and 5,000 units of IL-2 in patients 1(A) and 17,000 and 67,000 units in Patient 2(B). Points indicated are means$\pm$s.d. of triplicate samples of $10^5$ mononuclear cells. Uptake was measured over a 4-h culture period *in vitro* in the presence of 2$\mu$Ci of $^{125}$IUDR.

mononuclears was increased in blood samples taken immediately after IL-2 injection and returned to baseline levels by 24h. In Patient 1 there was no significant increase in $^{125}$IUDR uptake in samples taken from Day 1 after the first or second injection of IL-2. This also applied to Patient 2 after the first injection but there was a significant increase in $^{135}$IUDR incorporation in leukocytes taken 6, 7, 9 and 13 days after the second IL-2 injection.

*Haematological changes after i.v. injection of the partially purified IL-2 containing supernatants*

The haematological data obtained from sequential studies of the 2 patients after IL-2 administration is recorded in Table IV. The results can be summarized as follows: (1) No significant changes occurred in haemoglobin (Hb) levels in Patient 1 (who had metastases in bone marrow). In Patient 2 there was a slight increase in Hb at Days 7–12 after both injections. This increase coincided with an increase in platelet counts, and myelocytes and metamyelocytes. (2) The total white blood cell (WBC) count and the neutrophil (neut) count showed a marked increase 2–4h after the injections but returned to normal by Day 1 (1d) or Day 2 (2d). (3) Lymphocyte (lym) counts were reduced immediately after the injections. They returned to pre-treatment values 1 day after the injections in Patient 1. In Patient 2 the lymphocyte count tended

**Table IV** Haematological changes after injection of supernatants containing IL-2

Time after injection of IL-2

| *Patient 1* | 14,000 units | | | | | | | | 5,000 units | | | | | | |
|---|---|---|---|---|---|---|---|---|---|---|---|---|---|---|---|
| | 0 | 4h | 1d | 2d | 3d | 7d | 13d | 25d | 0 | 4h | 1d | 6d | 8d | 12d | 15d |
| Hb.[1] | 11.9 | 13.0 | 11.6 | 11.4 | 11.5 | 11.2 | 11.0 | 11.0 | 11.0 | 10.6 | 10.6 | 10.6 | 10.1 | 10.7 | |
| WBC[2] | 7.1 | 14.7 | 7.6 | 5.6 | 6.8 | 6.1 | 5.2 | 5.7 | 5.6 | 15.4 | 8.4 | 7.8 | 5.2 | 6.3 | 5.0 |
| Neut. | 5.6 | 13.2[4] | 6.0 | 3.9 | 5.8 | 4.9 | 4.2 | 4.0 | 4.2 | 14.3 | 7.2 | 5.8 | 4.0 | 5.2 | 4.0 |
| Lymph. | 0.7 | 0.4[3] | 0.98 | 1.1 | 0.5 | 0.48 | 0.57 | 1.2 | 0.7 | 0.46 | 0.9 | 1.3 | 0.7 | 0.6 | 0.6 |
| Mono. | 0.8 | 1.0 | 0.45 | 0.4 | 0.47 | 0.6 | 0.3 | 0.3 | 0.6 | 0.6 | 0.2 | 0.6 | 0.5 | 0.4 | 0.4 |
| Platelets | 326 | 289 | 347 | 317 | 332 | 412 | 439 | 341 | 360 | 345 | 381 | 404 | 445 | 451 | 339 |

| *Patient 2* | 17,000 units | | | | | | | 67,000 units | | | | | | | | | | | |
|---|---|---|---|---|---|---|---|---|---|---|---|---|---|---|---|---|---|---|---|
| | 0 | 4h | 1d | 2d | 7d | 9d | 12d | 0 | 4h | 1d | 2d | 3d | 7d | 8d | 9d | 12d | 16d | 21d | 23d |
| Hb. | 12.9 | 13.3 | 12.2 | 12.8 | 13.2 | 13.8 | 13.6 | 13.4 | 12.3 | 12.6 | 11.9 | 12.6 | 13.5 | 14.2 | 14.0 | 13.3 | 13.5 | 13.0 | 13.0 |
| WBC | 9.6 | 20.9 | 11.6 | 8.1 | 9.4 | 9.3 | 8.5 | 12.2 | 23.5 | 32.6 | 11.9 | 8.7 | 10.1 | 10.4 | 12.6 | 10.6 | 11.5 | 8.0 | 8.4 |
| Neut. | 7.1 | 19.6 | 10.3 | 7.0 | 7.5 | 7.9 | 7.4[4] | 9.5 | 22.8 | 30.0 | 10.5 | 7.5 | 7.3[4] | 8.8[4] | 10.8[4] | 9.1 | 10.5 | 6.1 | 6.9 |
| Lymph. | 2.1 | 0.63 | 1.04 | 0.81 | 1.4 | 1.3 | 0.77 | 1.59 | 0.5 | 1.3 | 0.8[3] | 1.2 | 2.2 | 0.9 | 1.1 | 0.8 | 0.7 | 1.5 | 1.2 |
| Mono. | 0.3 | 0.4 | 0.3 | 0.3 | 0.5 | — | 0.3 | 0.8 | 0.2 | 0.9 | 0.6 | 0.1 | 0.4 | 0.6 | 0.5 | 0.6 | 0.3 | 0.3 | 0.3 |
| MM & Myl. | — | — | — | — | — | — | — | — | — | — | — | — | occ. | 1 | occ. | — | — | — | — |
| Platelets | 328 | 295 | 312 | 324 | 404 | 472 | 471 | 443 | 383 | 377 | 337 | 371 | 430 | 518 | 508 | 483 | 477 | 369 | 373 |

[1] g/dl.
[2] Leukocyte and platelet counts $\times 10^{-9} l^{-1}$.
[3] Occasional atypical lymphocytes noted.
[4] Occasional hypersegmented neutrophils.

to remain below the initial pre-treatment values. In Patient 1 there was a secondary reduction of lymphocyte numbers after the first injection on Days 3, 7 and 13. In Patient 2 there also appeared to be secondary reduction in lymphocyte count on Day 2 following both injections and Days 12 and 16 after the second injection. (4) Monocyte (mono) counts showed no significant changes. (5) Platelet counts showed a reduction of 5–14% of pre-treatment counts by 4 h after the injections. This appeared related to the dose of IL-2 injected and persisted for 1–2 days.

*Clinical observations*

The following changes were observed clinically after i.v. injection of the IL-2 containing supernatants. Rigors developed ~10–15 min following injection and lasted for ½–1 h. This was followed by pyrexia at 38–39°C lasting from 2–6 h. The duration of the pyrexia was related to the number of IL-2 units injected. After 5,000 units the pyrexia lasted for ~2 h and after 67,000 units for 6 h. Tachycardia of 100–120 was noted during the pyrexia and a transient drop of blood pressure of 5–15 mmHg diastolic occurred at the beginning of the pyrexia. The first patient developed nausea and vomiting 15 min after the second injection of 5,000 units. The second patient developed transient nausea 10–15 min after the second injection of 67,000 units. Patient 1 was ambulant and attended for treatment as an outpatient. Patient 2 was an inpatient for stabilization of anti-coagulant therapy (given for a deep calf vein thrombosis which developed after his last surgical treatment) and for treatment of an ulcer over the lateral aspect of his left ankle. No clinically apparent adverse effects were noted in the days subsequent to the injections.

*Immunoglobulin levels* No significant change in immunoglobulins A, G or M levels were detected in serum samples from the 2 patients taken at weekly intervals over the study period (8–10 weeks).

*Auto-antibodies* Auto-antibodies to red blood cells, anti-nuclear factors, parietal cells, smooth muscle cells, thyroid tissue and mitochondria were not detected in pre-treatment serum samples or in samples taken ~3 weeks after each treatment. Rheumatoid factor was detected by the latex agglutination test both before and after treatment in Patient 2.

*Cortisol levels* An increase in cortisol levels were observed within 2 h of the injections. The degree of the rise appeared related to the dose of IL-2 given. In Patient 2 after injection of 17,000 units peak cortisol levels of 720 nM/l were seen at 4 h. After 67,000 units peak levels of 1780 nM/l were seen at 4 h. Pre-injection values were 280 and 270 nM/l respectively. The levels had returned to within the normal range 20 h after the injections (270 and 415 nM/l respectively. Normal range 280–830 nM/l).

*Liver enzymes and blood glucose levels* No significant change in the enzyme alkaline phosphatase, lactic dehydrogenase, glutamyl pyruvate, glutamyl oxalacetic and γ-glutamyl transaminases were recorded at 1, 4 and 24 h after the IL-2 injection. The serum glucose level in Patient 1 before and at 1, 4 and 24 h after injection of 14,000 units was 4.4, 3.3, 6.5 and 5.1 mM $l^{-1}$ respectively. (Normal venous fasting levels 3.9–6.1 mM $l^{-1}$). In Patient 2 levels of blood glucose before and at 1, 4 and 24 h after injection of 67,000 units was 5.3, 3.3, 5.3 and 5.8 respectively.

*Effect of IL-2 on the coagulation system* Patient 2 was on anti-coagulants and was not studied. At 45 min after injection of 14,000 units in Patient 1 there was significant prolongation of the thrombin time from 14 to 30 sec and of the partial thromboplastin time with kaolin (PTTK) of 27 to 35 sec. These had returned to normal by 4 h. Moderate levels of fibrinogen degradation products (FDPs) were detected at 45 min (40–80 ng $ml^{-1}$) and fibrinogen levels were also low 45 min after the injections (15 mg $ml^{-1}$) (Normal range, 200–400 mg/ml). The latter had returned to normal by 4 h but 10–40 ng $ml^{-1}$ of FDPs were still present at this time.

*IFN levels in serum following administration of IL-2 containing supernatants* No significant levels of IFN (<1 unit/ml) were detected in serum samples taken at 1 and 4 h or at the daily intervals shown in Figures 2 and 3 after injection of 14,000 units in Patient 1 and 67,000 units in Patient 2.

*Complement levels* No significant changes occurred in the $C_3$ or $C_4$ levels after the injections in either patient.

### Discussion

The dose of IL-2 given to the subjects in this study was selected so that an initial level of at least 2 u/ml in plasma would be obtained as this was the level used *in vitro* for induction of cytotoxic T cell activity (Hersey *et al.*, 1981). Based on an expected plasma volume of ~4% of body weight and assuming no destruction or absorption of the IL-2 it would be expected that 14,000 u in Patient 1 would give an initial value of 7.3 u $ml^{-1}$ (calculated plasma volume 1.92 l) and 17,000 u in Patient 2 an initial value of 5.8 u $ml^{-1}$ (calculated plasma volume 2.92 l). Observed values were approximately 3 u/ml and 1 u/ml respectively. Similarly on the assumption that IL-2 equilibrated in total extracellular fluid (TEF) and that this was 19% of body weight it would be expected that the final level in Patient 1 would be 1.5 u. $ml^{-1}$ (calculated TEF = 9.12 l) and 1.22 u. ml in Patient 2 (calculated TEF = 13.87 l). In fact zero levels were seen at 1 h. [Equivalent values for the second injection in both patients were Patient 1, expected initial 2.6 u $ml^{-1}$ (observed approximately 1.25 u/ml, Patient 2 expected initial 23 u/$ml^{-1}$, observed approximately 6 u $ml^{-1}$)].

The discrepancy between the expected and observed IL-2 values in the circulation may be the result of several factors. One possibility is the IL-2 may be inactivated by factors in serum as reported to occur in mice (Wagner *et al.*, 1980*a*). Similar inactivation of IL-2 by human serum was not detected in the present studies. The mitogenic activity of IL-2 on activated T cells *in vitro* was only inhibited by high concentrations of fresh human serum and the induction of cytotoxic cells *in vitro* by IL-2 in autologous serum was comparable or only moderately reduced compared to that in FCS. We cannot exclude that factors inhibiting mitogenesis may have been released into the serum *in vivo* following IL-2 administration and that these may have influenced the clearance rates by their effect on the bioassay used in the study.

A second explanation for loss of IL-2 activity is that it was absorbed by activated T cells *in vivo* as shown to occur *in vitro*. (Smith *et al.*, 1979; Watson & Mochizuki, 1980). At high cell concentrations *in vitro* 70% of IL-2 activity was found to be absorbed in 30 min (Smith *et al.*, 1979) which is comparable to the clearance rate from the patient's serum found in the present study. Absorption of IL-2 by activated lymphocytes may explain why different levels of IL-2 were seen in the 2 patients for a given dose of IL-2. Patient 1 had a lower lymphocyte count than Patient 2 and hence less absorption of IL-2 and relatively higher serum levels of IL-2 may have occurred.

The latter explanation was supported by evidence of increased division of cells in the circulation after injection of the IL-2 containing preparations. This was consistent with absorption to and activation of cells in the circulation which expressed IL-2 receptors. Recent studies suggest the latter cells are activated into the $G_1$ phase of the cell cycle by interaction with antigen but require IL-2 for subsequent cell division (Kristensen *et al.*, 1982).

A number of clinical and biochemical effects were observed in the recipients of the IL-2 containing preparations which may have reflected the activity of contaminating monokines and lymphokines in the preparation, e.g. pyrexia may have resulted from interleukin I (IL-1) in the supernatants as the latter is believed to be synonymous with endogenous pyrogen (Murphy *et al.*, 1980). Similarly the transient hypoglycaemia and increase in FDPs may indicate the activity of such factors as glucocorticoid antagonizing factor (Moore *et al.*, 1978) and thromboplastins (Geczy & Hopper, 1981) released from monocytes in the blood leukocyte preparations during IL-2 production. The increased platelet count, haemoglobin and metamyelocytes noted at 7 days may indicate the presence of colony stimulating factors in the preparations.

Elevation of glucocorticoids in the circulation was previously noted after injection of lymphokine preparations in rodents (Besedovsky *et al.*, 1981) and may have been responsible in part for the neutrophilia and lymphocytopenia noted hours after the injections. Both the latter are well known systemic effects of corticosteroids. The systemic effects noted in these studies were similar to those reported after administration of a lymphokine preparation (Dumonde *et al.*, 1982) and recombinant IFN except that neutropenia rather than neutrophilia was noted after the latter (Gutterman *et al.*, 1982). IFN was not detected in the IL-2 containing supernatants in the present study and it seems likely that the side effects common to these procedures are in part due to a stress response.

These results highlight the need for administration of purified preparations of IL-2 to allow the effects of IL-2 *in vivo* to be clearly defined. The present study, however, provides some encouragement that systemic administration of IL-2 may be a feasible approach for the activation of T cells *in vivo*. Such an approach would be technically much simpler than injection of cytotoxic T cells grown *in vitro* in the presence of IL-2 as described by previous authors (Mills *et al.*, 1980; Warren *et al.*, 1979; Gillis & Watson, 1981) and may allow activation of lymphocytes at the site of tumour growth. Although the half-life of 20–25 min for IL-2 in the circulation appears relatively short this does not appear to be due to inactivation by serum factors. If subsequent studies confirm that clearance is at least in part due to absorption to activated T cells this may be sufficient to trigger these cells into cytotoxic activity despite the subsequent absence of detectable IL-2 in the circulation. Further studies with purified IL-2 preparations are required to answer these questions.

This work was supported in part by NCI contract NO1-CB-74120, the Department of Surgery, University of Sydney and Melanoma Research Fund, Sydney Hospital. We wish to thank Sister J. Gardner for collection of clinical specimens and the volunteers who donated their leukocytes for the study. We also wish to thank Dr. S. Deveridge and the trained nursing staff who conducted leukopheresis on the volunteer donors. We are grateful to the Haematology and Biochemistry Departments of the Kanematsu Memorial Institute for carrying out the coagulation studies and assay of blood glucose, liver enzyme, corticosteroid and complement levels.

### References

BESEDOVSKY, H.O., DELREY, A. & SORKIN, E. (1981). Lymphokine-containing supernatants from Con A-stimulated cells increase corticosterone blood levels. *J. Immunol.*, **126**, 385.

DUMONDE, D.C., PULLEY, M.S., HAMBLIN, A.S. & OTHERS (1981). In: *Lymphokines and Thymic Hormones: Their Potential Utilization in Cancer Therapeutics*. (Eds. Goldstein & Chirigos) New York: Raven Press, p. 00.

GECZY, C.L. & HOPPER, K.E. (1981). A mechanism of migration inhibition in delayed-type hypersensitivity reactions. II. Lymphokines promote procoagulant activity of macrophages *in vitro*. *J. Immunol.*, **126**, 1059.

GILLIS, S. & WATSON, J. (1981). Interleukin 2 dependent culture of cytotoxic T cell lines. *Immunol. Rev.*, **54**, 81.

GUTTERMAN, J.U., FEIN, S., QUESADA, J. & 10 OTHERS (1982). *Ann. Int. Med.* (In press).

HERSEY, P., BINDON, C., EDWARDS, A., MURRAY, E., PHILLIPS, G. & McCARTHY, W.H. (1981). Induction of cytoxic activity in human lymphocytes against autologous and allogeneic melanoma cells *in vitro* by culture with interleukin 2. *Int. J. Cancer*, **28**, 695.

HERSEY, P., EDWARDS, A., LEWIS, R., SINGH, S., KEMP, A. & McINNIS, J. (1982). Deficient natural killer cell activity in a patient with Fanconi's anaemia and squamous cell carcinoma. Association with defect in interferon release. *Clin. Exp. Immunol.*, **48**, 205.

KRISTENSEN, F., WALKER, C., BETTENS, F., JONCOURT, F. & de WECK, A.L. (1982). Assessment of IL-1 and IL-2 effects on cycling and non-cycling murine thymocytes. *J. Immunol.* (In press).

LAFFERTY, K.J., ANDRUS, L. & PROWSE, S.J. (1980). Role of lymphokine and antigen in the control of specific T cell responses. *Immunol. Rev.*, **51**, 279.

LOTZE, M.T., LINE, B.B., MATHISEN, D.J. & ROSENBERG, S.A. (1980). The *in vivo* distribution of autologous human and murine lymphoid cells grown in T cell growth factors (TCGF): Implications for the adoptive immunotherapy of tumors. *J. Immunol.*, **125**, 1487.

MIER, J.W. & GALLO, R.C. (1980). Purification and some characteristics of human T-cell growth factor from phytohemagglutinin-stimulated lymphocyte-conditioned media. *Proc. Natl Acad. Sci.* **77**, 6134.

MILLS, G.B. & PAETKAU, V. (1980). Generation of cytotoxic lymphocytes to syngeneic tumor by using co-stimulator (interleukin 2). *J. Immunol.*, **125**, 1897.

MILLS, G.B., CARLSON, G. & PAETKAU, V. (1980). Generation of cytotoxic lymphocytes to syngeneic tumors by using co-stimulator (interleukin 2): *in vivo* activity. *J. Immunol.*, **125**, 1904.

MOORE, R.N., GOODRUM, K.J., COUCH, R. & BERRY, L.J. (1978). Factors affecting macrophage function: glucocorticoid antagonizing factor. RES. *J. Reticuloendothel. Soc.*, **23**, 321.

MURPHY, P.A., SIMON, P.L. & WILLOUGHBY, W.F. (1980). Endogenous pyrogens made by peritoneal exudate cells are identical with lymphocyte activating factors made by rabbit alveolar macrophages. *J. Immunol.*, **124**, 2498.

SMITH, K.A., GILLIS, S., BAKER, P.E., McKENZIE, D., RUSCETTI, F.W. (1979). T-cell growth factor-mediated T-cell proliferation. *Ann. N.Y. Acad. Sci.*, **332**, 423.

WAGNER, H., HARDT, C., HEEG, K. & OTHERS (1980*a*). T-T interactions during cytotoxic T lymphocyte (CTL) responses: T-cell derived helper factor (interleukin 2) as a probe to analyse CTL responsiveness and thymic maturation of CTL progenitors. *Immunol. Rev.*, **51**, 215.

WAGNER, H., HARDT, C., HEEG, K., ROLLINGHOF, M. & PFIZENMAIER, K. (1980*b*). T-cell derived helper factors allows *in vivo* induction of cytotoxic T cells in nu/nu mice. *Nature*, **284**, 278.

WARREN, H.S., WOOLNOUGH, J.A. & LAFFERTY, K.J. (1978). Cytotoxic T cell responses to a syngeneic tumor. Conditions for primary activation *in vitro*. *Aust. J. Exp. Biol. Med. Sci.*, **56**, 247.

WARREN, H.S., WOOLNOUGH, J.A., DENARO, C.P. & LAFFERTY, K.J. (1979). Cytotoxic T cell responses to a syngeneic tumor: Conditions for primary activation *in vitro* and biological activity *in vivo*. *Adv. Exp. Med. Biol.*, **114**, 757.

WATSON, J. & MOCHIZUKI, D. (1980). Interleukin 2: A class of T-cell growth factors. *Immunol. Rev.*, **51**, 257.

VOSE, B.M. & BONNARD, G.D. (1982). Specific cytotoxic and proliferative responses of human lymphocytes grown in IL-2 against autologous tumor. *Int. J. Cancer*, **29**, 33.

VOSE, B.M. & MOORE, M. (1981). Cultured human T cell lines kill autologous solid tumors. *Immunol. Letters*, **3**, 237.

ZARLING, J.M. & BACH, F.H. (1979). Continuous culture of T cells cytotoxic for autologous human leukemia cells. *Nature*, **280**, 685.